# Retraction: *Porphyromonas gingivalis* promotes progression of esophageal squamous cell cancer via TGFβ-dependent Smad/YAP/TAZ signaling

**DOI:** 10.1371/journal.pbio.3001617

**Published:** 2022-04-15

**Authors:** The PLOS Biology Editors

Following the publication of this article [1], the corresponding author contacted the journal to state that errors had occurred in the preparation of Fig 1, Fig 3 and S4 Fig. Additional concerns were raised about other results reported in the article, and about data provided to PLOS in follow-up discussions.

Specifically,

1. Similarities were noted between images representing different experimental results. This affects results reported in 14 panels of Figs 1E, 1F, 3B, 4E, S3D, S4F, and S4G.
2. In S2H Fig., the following tumors appear similar despite being used to represent different experimental conditions: *P*. *gingivalis* (3rd tumor from left) and *P*. *gingivalis* + Tinidazole (1st tumor from left); *P*. *gingivalis* + Tinidazole (5th tumor from left) and *P*. *gingivalis* + SB-431542 (4th tumor from left).
3. In S4B Fig., the KYSE30 siCON + *P*. *gingivalis* Smad2/3 panel does not appear to match the corresponding Merge panel despite representing the same sample and field of view. Similarly, the zoomed panels for red fluorescence and Merge panels do not appear to match each other for several results reported in Fig 3B and S3C Fig.
4. The following blot results do not appear to match the corresponding data provided in the S1 Raw Images file:
  - Fig 2F: NE-6 and KYSE30 Smad2/3 panels.
  - Fig 3C: KYSE30 pYAP and GAPDH panels.
5. Based on raw data provided by the corresponding author post-publication, tumor volumes for graphs presented in Figs 2H and 4F, and S2H Fig. were calculated inconsistently within each data set. Furthermore, tumor size data indicated that several tumors exceeded internationally-accepted limits for rodent tumor experiments, and several images indicated that mice had large, ulcerated tumors at the end of the experiments. The tumor data raised concerns about animal welfare and the adherence of the study to animal research ethics standards.

The corresponding author confirmed that several errors were introduced during figure preparation. They provided data underlying the results in question, which depending on the figure consisted of the original data, data from replicate experiments, or replacement data. The corresponding author noted that the original raw data are no longer available for the Fig 2F, NE-6 Smad2/3 panel, and Fig 3C, KYSE30 pYAP and GAPDH panels; replicate data were provided for these panels.

In addition to the issues identified during editorial assessment, the corresponding author stated that the S3C Fig. NE6-T siSmad2/3 YAP panel, and the S4F Fig. Input GAPDH (top panel) were incorrect and provided replacement images.

For S2H Fig., in which there were concerns about multiple tumor image duplications, the corresponding author stated that incorrect tumor images were used and provided replacement images showing different tumors for all conditions.

After careful consideration of the extent of image issues and the comments received in response, *PLOS Biology* concluded that the extent of data reporting issues exceeds what is appropriate to be addressed via a Correction, and calls into question the overall reliability of the reported results and conclusions. In light of this overarching issue and the animal research concerns discussed below, *PLOS Biology* did not undertake a detailed review of the replacement data provided.

In response to point 4, the corresponding author confirmed that calculation of tumor volume in Figs 2H and 4F, and S2H Fig. was performed inconsistently. They provided re-analysis of tumor volumes using the original data, as well as tumor data from replicate experiments.

The corresponding author also commented that internationally-accepted standards were followed for all tumor studies, and all mice were euthanized when any tumor exceeded 20mm in diameter, or 2000mm^3^ in volume, or if ulcers, inflammation or necrosis were observed. They stated that animal welfare was checked every two days, and no animals exhibited the humane endpoints described above two days before the final welfare check and euthanasia. Time course data confirmed tumors exceeding the above limits were observed only at the final timepoint. The size and condition of tumors pictured in the underlying data call into question whether the monitoring frequency was sufficient to mitigate animal welfare concerns in the reported experiments.

A representative of the Research Ethics Committee of the First Affiliated Hospital of Henan University of Science and Technology stated that IACUC and IRB applications for this study were submitted in 2015 and approved in January 2016. Nevertheless, the comments, data, and documentation received in response to point 5 did not fully resolve PLOS’ concerns about animal research ethics and animal welfare considerations in the tumor studies. Questions remain about the article’s compliance with the PLOS Animal Research policy which requires that, “Studies involving animals must be conducted according to internationally-accepted standards.”

Due to the above concerns about the reliability of the article’s results and conclusions, and the study’s compliance with the PLOS Animal Research Policy, the *PLOS Biology* Editors retract this article. We regret that the issues in this article were not identified prior to publication.

All authors noted that they did not agree with the retraction, stand by the article’s findings, and apologize for the issues with the published article.
